# Aging and Emotion: Regulation Tactics Alter Self-Reported and Physiological Arousal

**DOI:** 10.1093/geroni/igaf122.3534

**Published:** 2025-12-31

**Authors:** Ceresa Munjak-Khoury, Rebecca Polk, Derek Isaacowitz

**Affiliations:** Washington University in St. Louis, St. Louis, Missouri, United States; Washington University in St. Louis, St Louis, Missouri, United States; Washington University in St. Louis, St. Louis, Missouri, United States

## Abstract

Older adults consistently report higher emotional well-being despite some physical and mental declines with age. Some theorize this is due to differences in emotion regulation, however no conclusive evidence for age differences in emotion regulation strategies has emerged. Emotion regulation tactics, such as positive-approaching (enhancing the positivity of a situation) and negative-receding (reducing the negativity of a situation), hold promise for uncovering age differences. However, no studies to date have considered whether tactic vary in their physiological profiles. Thus, this study investigated 35 younger (M = 19.06 years, SD = 3.58; 17 women) and 42 older (M = 74.02 years, SD = 4.69; 23 women) participants who viewed emotionally-evocative videos and regulated emotions using positive-approaching and negative-receding techniques (with both regulation blocks compared to a neutral video block to control for baseline differences in psychophysiology). Multi-level linear models revealed significant Age x Sex x Tactic interactions, such that positive-approaching tactics (vs negative-receding) were associated with lower arousal ratings for younger women and older men (but not younger men or older women). While there was no tactic difference in self-reported arousal for older women, Respiratory Sinus Arrythmia (a measure of heart activity associated with calm parasympathetic states) was higher during positive-approaching for them. Interestingly, older men had higher RSA during negative-approaching tactics as compared to positive-approaching. These findings suggest emotion regulation tactics have age and sex differential impacts on self-reported and physiological arousal. Future work may disentangle physiological activation and self-reported behavior by also considering the role of interoceptive awareness.

